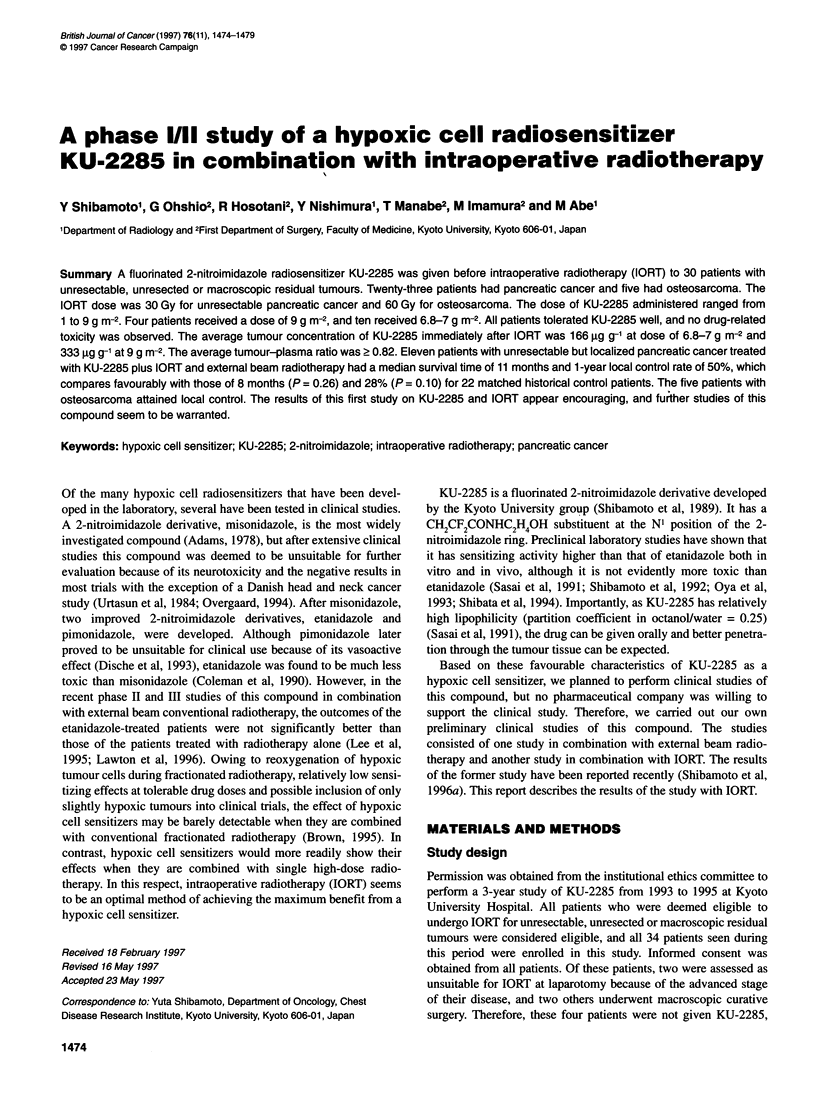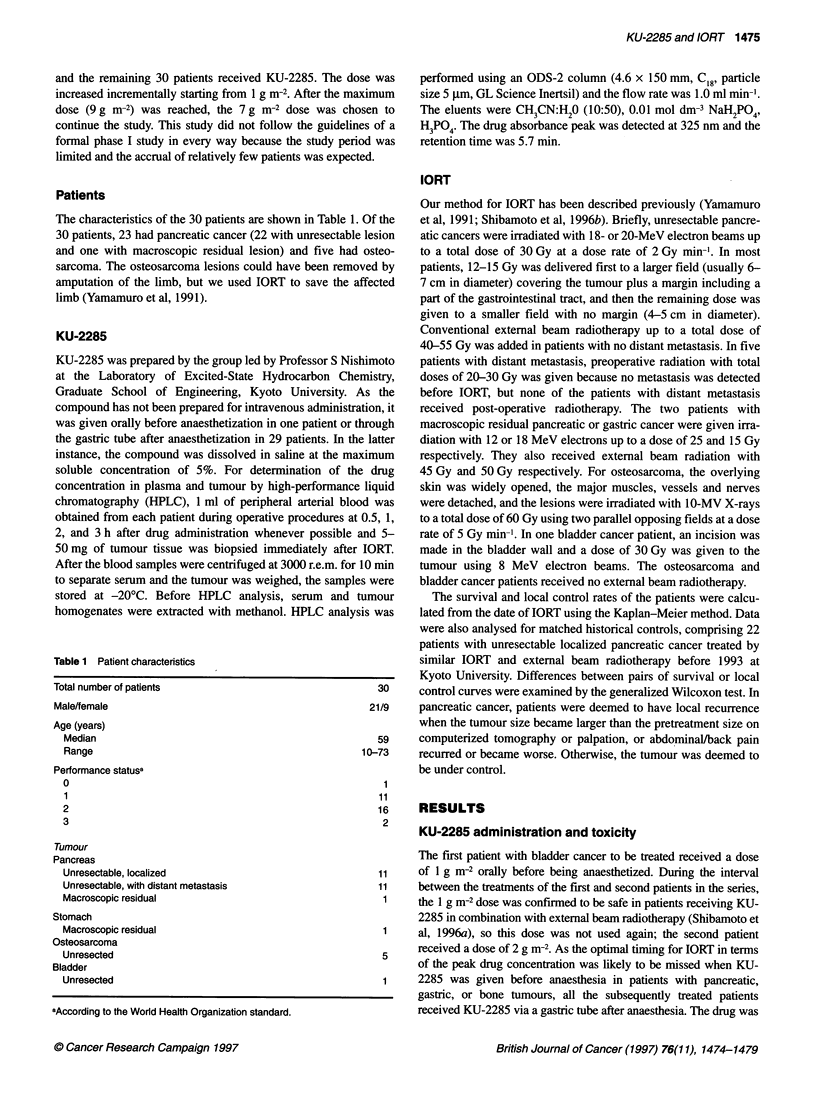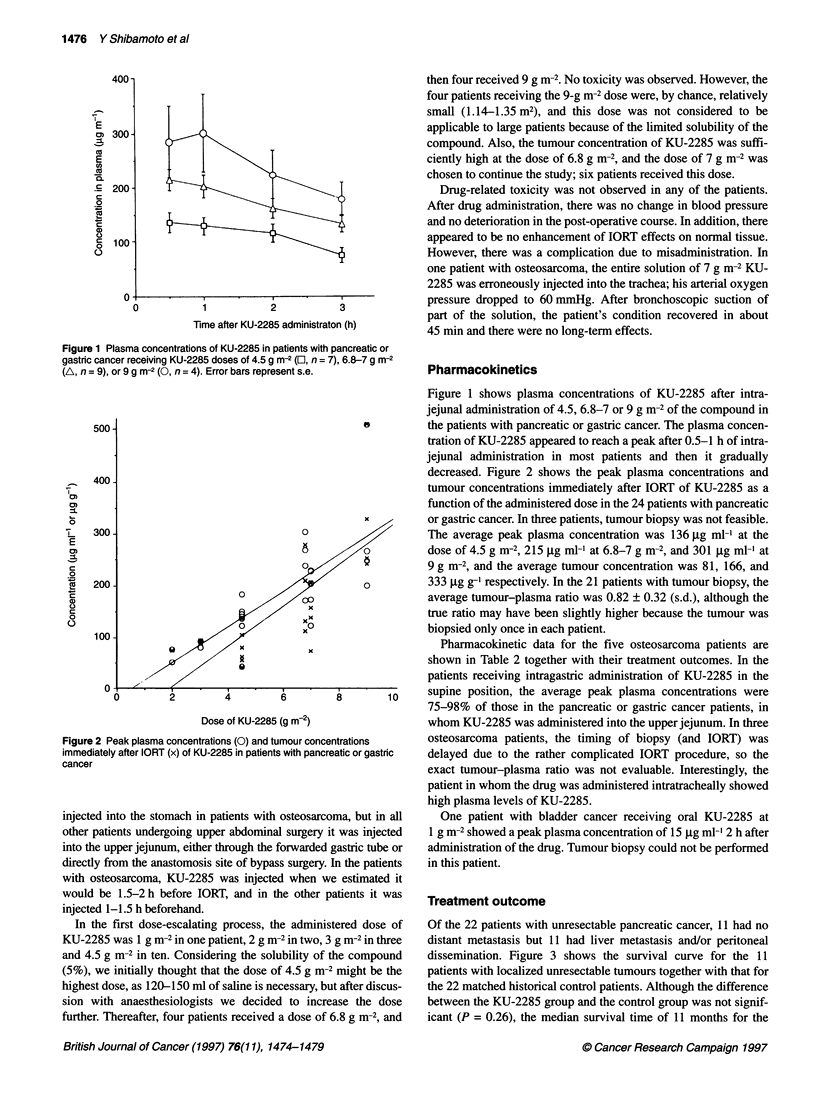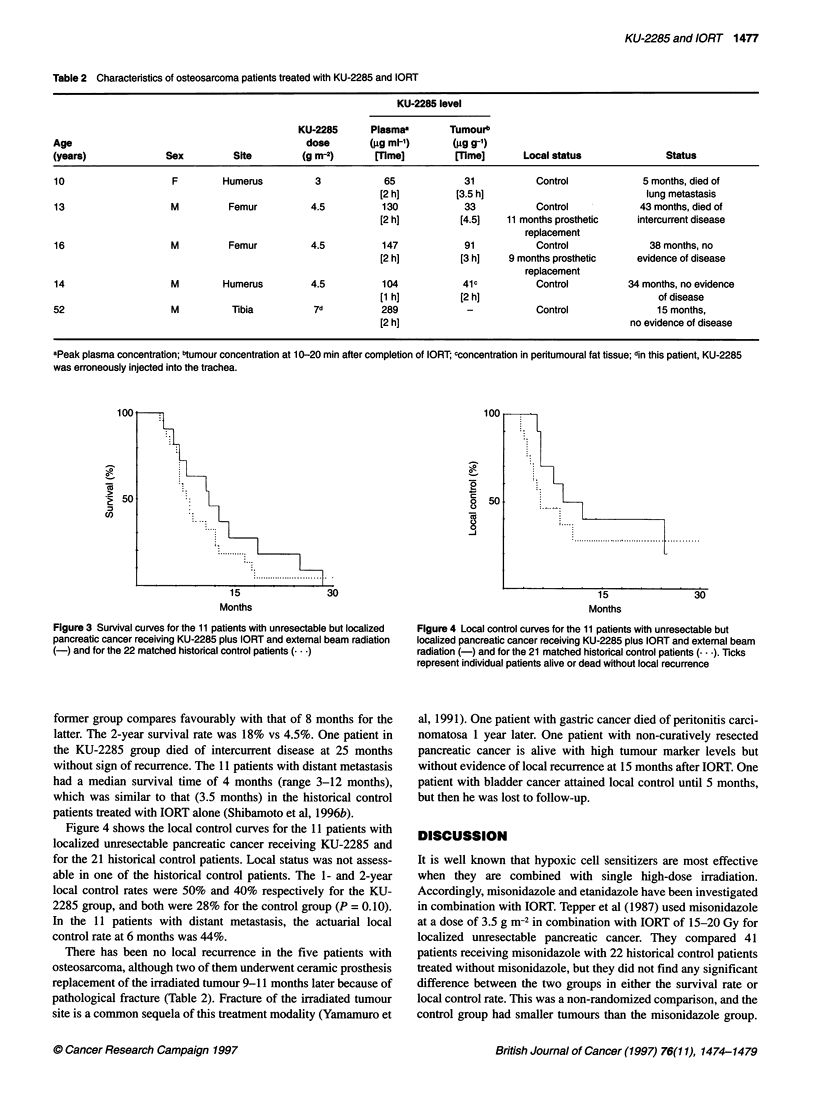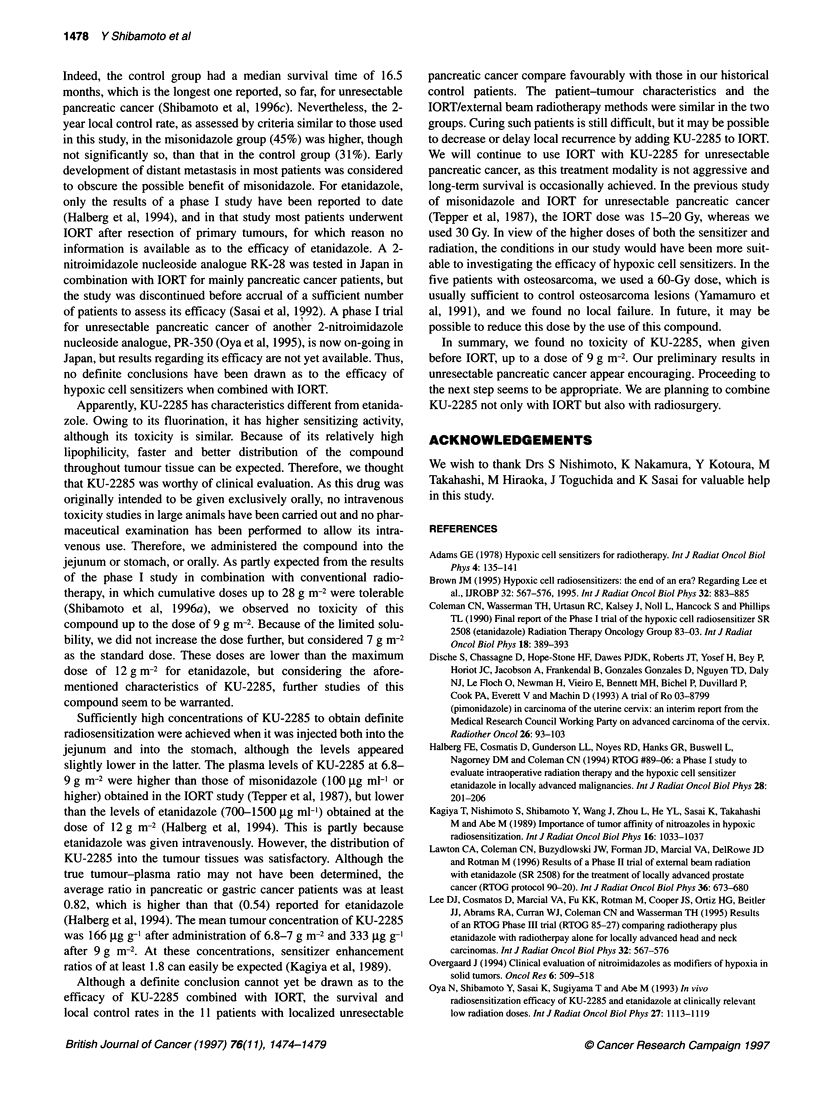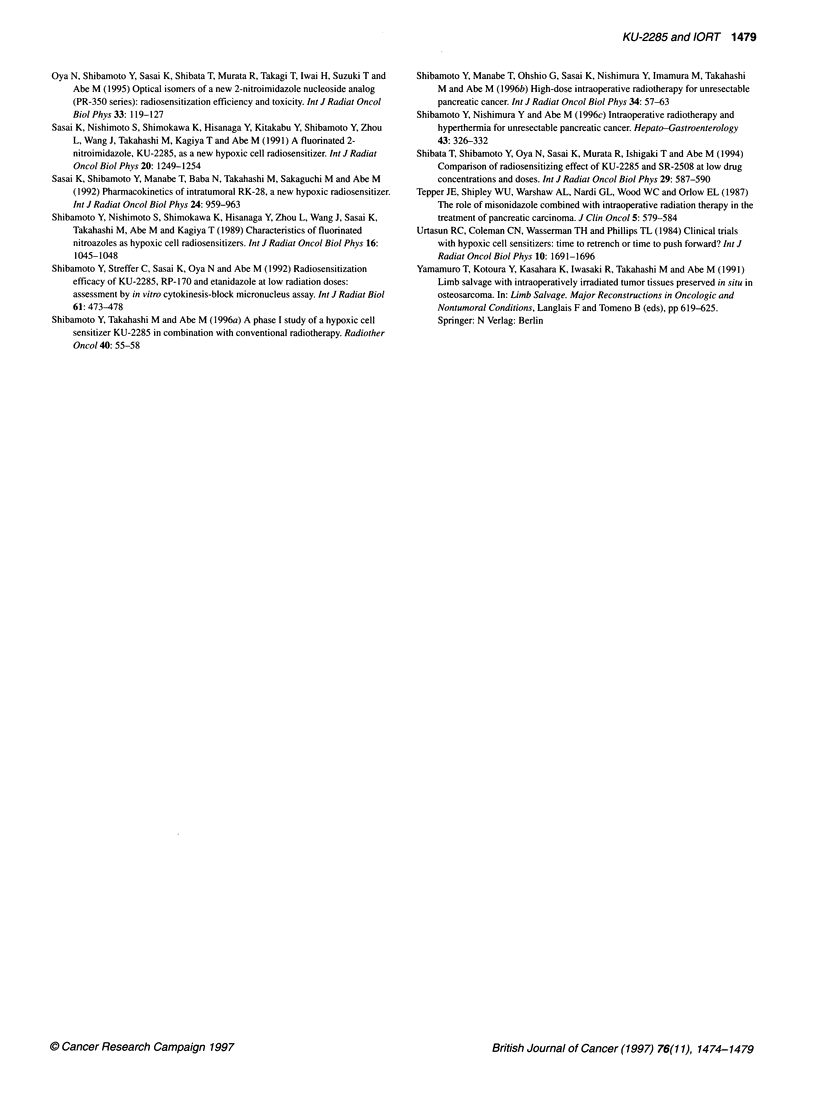# A phase I/II study of a hypoxic cell radiosensitizer KU-2285 in combination with intraoperative radiotherapy.

**DOI:** 10.1038/bjc.1997.580

**Published:** 1997

**Authors:** Y. Shibamoto, G. Ohshio, R. Hosotani, Y. Nishimura, T. Manabe, M. Imamura, M. Abe

**Affiliations:** Department of Radiology, Faculty of Medicine, Kyoto University, Japan.

## Abstract

A fluorinated 2-nitroimidazole radiosensitizer KU-2285 was given before intraoperative radiotherapy (IORT) to 30 patients with unresectable, unresected or macroscopic residual tumours. Twenty-three patients had pancreatic cancer and five had osteosarcoma. The IORT dose was 30 Gy for unresectable pancreatic cancer and 60 Gy for osteosarcoma. The dose of KU-2285 administered ranged from 1 to 9 g m-2. Four patients received a dose of 9 g m-2, and ten received 6.8-7 g m-2. All patients tolerated KU-2285 well, and no drug-related toxicity was observed. The average tumour concentration of KU-2285 immediately after IORT was 166 microg g-1 at dose of 6.8-7 g m-2 and 333 microg g-1 at 9 g m-2. The average tumour-plasma ratio was > or = 0.82. Eleven patients with unresectable but localized pancreatic cancer treated with KU-2285 plus IORT and external beam radiotherapy had a median survival time of 11 months and 1-year local control rate of 50%, which compares favourably with those of 8 months (P = 0.26) and 28% (P = 0.10) for 22 matched historical control patients. The five patients with osteosarcoma attained local control. The results of this first study on KU-2285 and IORT appear encouraging, and further studies of this compound seem to be warranted.


British Journal of Cancer (1997) 76(11), 1474–1479
© 1997 Cancer Research Campaign

### A phase I/II study of a hypoxic cell radiosensitizer KU-2285 in combination with intraoperative radiotherapy

**Y Shibamoto[1], G Ohshio[2], R Hosotani[2], Y Nishimura[1], T Manabe[2], M Imamura[2] and M Abe[1]**

[1]Department of Radiology and [2]First Department of Surgery, Faculty of Medicine, Kyoto University, Kyoto 606-01, Japan

**Summary** A fluorinated 2-nitroimidazole radiosensitizer KU-2285 was given before intraoperative radiotherapy (IORT) to 30 patients with unresectable, unresected or macroscopic residual tumours. Twenty-three patients had pancreatic cancer and five had osteosarcoma. The IORT dose was 30 Gy for unresectable pancreatic cancer and 60 Gy for osteosarcoma. The dose of KU-2285 administered ranged from 1 to 9 g $m^{-2}$. Four patients received a dose of 9 g $m^{-2}$, and ten received 6.8–7 g $m^{-2}$. All patients tolerated KU-2285 well, and no drug-related toxicity was observed. The average tumour concentration of KU-2285 immediately after IORT was 166 μg $g^{-1}$ at dose of 6.8–7 g $m^{-2}$ and 333 μg $g^{-1}$ at 9 g $m^{-2}$. The average tumour–plasma ratio was ≥ 0.82. Eleven patients with unresectable but localized pancreatic cancer treated with KU-2285 plus IORT and external beam radiotherapy had a median survival time of 11 months and 1-year local control rate of 50%, which compares favourably with those of 8 months ($P$ = 0.26) and 28% ($P$ = 0.10) for 22 matched historical control patients. The five patients with osteosarcoma attained local control. The results of this first study on KU-2285 and IORT appear encouraging, and further studies of this compound seem to be warranted.

**Keywords:** hypoxic cell sensitizer; KU-2285; 2-nitroimidazole; intraoperative radiotherapy; pancreatic cancer

Of the many hypoxic cell radiosensitizers that have been developed in the laboratory, several have been tested in clinical studies. A 2-nitroimidazole derivative, misonidazole, is the most widely investigated compound (Adams, 1978), but after extensive clinical studies this compound was deemed to be unsuitable for further evaluation because of its neurotoxicity and the negative results in most trials with the exception of a Danish head and neck cancer study (Urtasun et al, 1984; Overgaard, 1994). After misonidazole, two improved 2-nitroimidazole derivatives, etanidazole and pimonidazole, were developed. Although pimonidazole later proved to be unsuitable for clinical use because of its vasoactive effect (Dische et al, 1993), etanidazole was found to be much less toxic than misonidazole (Coleman et al, 1990). However, in the recent phase II and III studies of this compound in combination with external beam conventional radiotherapy, the outcomes of the etanidazole-treated patients were not significantly better than those of the patients treated with radiotherapy alone (Lee et al, 1995; Lawton et al, 1996). Owing to reoxygenation of hypoxic tumour cells during fractionated radiotherapy, relatively low sensitizing effects at tolerable drug doses and possible inclusion of only slightly hypoxic tumours into clinical trials, the effect of hypoxic cell sensitizers may be barely detectable when they are combined with conventional fractionated radiotherapy (Brown, 1995). In contrast, hypoxic cell sensitizers would more readily show their effects when they are combined with single high-dose radiotherapy. In this respect, intraoperative radiotherapy (IORT) seems to be an optimal method of achieving the maximum benefit from a hypoxic cell sensitizer.

*Received 18 February 1997*
*Revised 16 May 1997*
*Accepted 23 May 1997*

*Correspondence to:* Yuta Shibamoto, Department of Oncology, Chest Disease Research Institute, Kyoto University, Kyoto 606-01, Japan

KU-2285 is a fluorinated 2-nitroimidazole derivative developed by the Kyoto University group (Shibamoto et al, 1989). It has a $CH_2CF_2CONHC_2H_4OH$ substituent at the $N^1$ position of the 2-nitroimidazole ring. Preclinical laboratory studies have shown that it has sensitizing activity higher than that of etanidazole both in vitro and in vivo, although it is not evidently more toxic than etanidazole (Sasai et al, 1991; Shibamoto et al, 1992; Oya et al, 1993; Shibata et al, 1994). Importantly, as KU-2285 has relatively high lipophilicity (partition coefficient in octanol/water = 0.25) (Sasai et al, 1991), the drug can be given orally and better penetration through the tumour tissue can be expected.

Based on these favourable characteristics of KU-2285 as a hypoxic cell sensitizer, we planned to perform clinical studies of this compound, but no pharmaceutical company was willing to support the clinical study. Therefore, we carried out our own preliminary clinical studies of this compound. The studies consisted of one study in combination with external beam radiotherapy and another study in combination with IORT. The results of the former study have been reported recently (Shibamoto et al, 1996*a*). This report describes the results of the study with IORT.

### MATERIALS AND METHODS

### Study design

Permission was obtained from the institutional ethics committee to perform a 3-year study of KU-2285 from 1993 to 1995 at Kyoto University Hospital. All patients who were deemed eligible to undergo IORT for unresectable, unresected or macroscopic residual tumours were considered eligible, and all 34 patients seen during this period were enrolled in this study. Informed consent was obtained from all patients. Of these patients, two were assessed as unsuitable for IORT at laparotomy because of the advanced stage of their disease, and two others underwent macroscopic curative surgery. Therefore, these four patients were not given KU-2285, and the remaining 30 patients received KU-2285. The dose was increased incrementally starting from 1 g $m^{-2}$. After the maximum dose (9 g $m^{-2}$) was reached, the 7 g $m^{-2}$ dose was chosen to continue the study. This study did not follow the guidelines of a formal phase I study in every way because the study period was limited and the accrual of relatively few patients was expected.

### Patients

The characteristics of the 30 patients are shown in Table 1. Of the 30 patients, 23 had pancreatic cancer (22 with unresectable lesion and one with macroscopic residual lesion) and five had osteosarcoma. The osteosarcoma lesions could have been removed by amputation of the limb, but we used IORT to save the affected limb (Yamamuro et al, 1991).

### KU-2285

KU-2285 was prepared by the group led by Professor S Nishimoto at the Laboratory of Excited-State Hydrocarbon Chemistry, Graduate School of Engineering, Kyoto University. As the compound has not been prepared for intravenous administration, it was given orally before anaesthetization in one patient or through the gastric tube after anaesthetization in 29 patients. In the latter instance, the compound was dissolved in saline at the maximum soluble concentration of 5%. For determination of the drug concentration in plasma and tumour by high-performance liquid chromatography (HPLC), 1 ml of peripheral arterial blood was obtained from each patient during operative procedures at 0.5, 1, 2, and 3 h after drug administration whenever possible and 5–50 mg of tumour tissue was biopsied immediately after IORT. After the blood samples were centrifuged at 3000 r.e.m. for 10 min to separate serum and the tumour was weighed, the samples were stored at –20°C. Before HPLC analysis, serum and tumour homogenates were extracted with methanol. HPLC analysis was performed using an ODS-2 column (4.6 × 150 mm, $C_{18}$, particle size 5 µm, GL Science Inertsil) and the flow rate was 1.0 ml $min^{-1}$. The eluents were $CH_3CN:H_20$ (10:50), 0.01 mol $dm^{-3}$ $NaH_2PO_4$, $H_3PO_4$. The drug absorbance peak was detected at 325 nm and the retention time was 5.7 min.

**Table 1** Patient characteristics

| | |
|---|---|
| Total number of patients | 30 |
| Male/female | 21/9 |
| Age (years) | |
| Median | 59 |
| Range | 10–73 |
| Performance status[a] | |
| 0 | 1 |
| 1 | 11 |
| 2 | 16 |
| 3 | 2 |
| *Tumour* | |
| Pancreas | |
| Unresectable, localized | 11 |
| Unresectable, with distant metastasis | 11 |
| Macroscopic residual | 1 |
| Stomach | |
| Macroscopic residual | 1 |
| Osteosarcoma | |
| Unresected | 5 |
| Bladder | |
| Unresected | 1 |

[a]According to the World Health Organization standard.

### IORT

Our method for IORT has been described previously (Yamamuro et al, 1991; Shibamoto et al, 1996*b*). Briefly, unresectable pancreatic cancers were irradiated with 18- or 20-MeV electron beams up to a total dose of 30 Gy at a dose rate of 2 Gy $min^{-1}$. In most patients, 12–15 Gy was delivered first to a larger field (usually 6–7 cm in diameter) covering the tumour plus a margin including a part of the gastrointestinal tract, and then the remaining dose was given to a smaller field with no margin (4–5 cm in diameter). Conventional external beam radiotherapy up to a total dose of 40–55 Gy was added in patients with no distant metastasis. In five patients with distant metastasis, preoperative radiation with total doses of 20–30 Gy was given because no metastasis was detected before IORT, but none of the patients with distant metastasis received post-operative radiotherapy. The two patients with macroscopic residual pancreatic or gastric cancer were given irradiation with 12 or 18 MeV electrons up to a dose of 25 and 15 Gy respectively. They also received external beam radiation with 45 Gy and 50 Gy respectively. For osteosarcoma, the overlying skin was widely opened, the major muscles, vessels and nerves were detached, and the lesions were irradiated with 10-MV X-rays to a total dose of 60 Gy using two parallel opposing fields at a dose rate of 5 Gy $min^{-1}$. In one bladder cancer patient, an incision was made in the bladder wall and a dose of 30 Gy was given to the tumour using 8 MeV electron beams. The osteosarcoma and bladder cancer patients received no external beam radiotherapy.

The survival and local control rates of the patients were calculated from the date of IORT using the Kaplan–Meier method. Data were also analysed for matched historical controls, comprising 22 patients with unresectable localized pancreatic cancer treated by similar IORT and external beam radiotherapy before 1993 at Kyoto University. Differences between pairs of survival or local control curves were examined by the generalized Wilcoxon test. In pancreatic cancer, patients were deemed to have local recurrence when the tumour size became larger than the pretreatment size on computerized tomography or palpation, or abdominal/back pain recurred or became worse. Otherwise, the tumour was deemed to be under control.

### RESULTS

### KU-2285 administration and toxicity

The first patient with bladder cancer to be treated received a dose of 1 g $m^{-2}$ orally before being anaesthetized. During the interval between the treatments of the first and second patients in the series, the 1 g $m^{-2}$ dose was confirmed to be safe in patients receiving KU-2285 in combination with external beam radiotherapy (Shibamoto et al, 1996*a*), so this dose was not used again; the second patient received a dose of 2 g $m^{-2}$. As the optimal timing for IORT in terms of the peak drug concentration was likely to be missed when KU-2285 was given before anaesthesia in patients with pancreatic, gastric, or bone tumours, all the subsequently treated patients received KU-2285 via a gastric tube after anaesthesia. The drug was

© Cancer Research Campaign 1997

**Figure 1** Plasma concentrations of KU-2285 in patients with pancreatic or gastric cancer receiving KU-2285 doses of 4.5 g m$^{-2}$ (□, $n$ = 7), 6.8–7 g m$^{-2}$ (△, $n$ = 9), or 9 g m$^{-2}$ (○, $n$ = 4). Error bars represent s.e.

**Figure 2** Peak plasma concentrations (○) and tumour concentrations immediately after IORT (x) of KU-2285 in patients with pancreatic or gastric cancer injected into the stomach in patients with osteosarcoma, but in all other patients undergoing upper abdominal surgery it was injected into the upper jejunum, either through the forwarded gastric tube or directly from the anastomosis site of bypass surgery. In the patients with osteosarcoma, KU-2285 was injected when we estimated it would be 1.5–2 h before IORT, and in the other patients it was injected 1–1.5 h beforehand.

In the first dose-escalating process, the administered dose of KU-2285 was 1 g m$^{-2}$ in one patient, 2 g m$^{-2}$ in two, 3 g m$^{-2}$ in three and 4.5 g m$^{-2}$ in ten. Considering the solubility of the compound (5%), we initially thought that the dose of 4.5 g m$^{-2}$ might be the highest dose, as 120–150 ml of saline is necessary, but after discussion with anaesthesiologists we decided to increase the dose further. Thereafter, four patients received a dose of 6.8 g m$^{-2}$, and then four received 9 g m$^{-2}$. No toxicity was observed. However, the four patients receiving the 9-g m$^{-2}$ dose were, by chance, relatively small (1.14–1.35 m$^2$), and this dose was not considered to be applicable to large patients because of the limited solubility of the compound. Also, the tumour concentration of KU-2285 was sufficiently high at the dose of 6.8 g m$^{-2}$, and the dose of 7 g m$^{-2}$ was chosen to continue the study; six patients received this dose.

Drug-related toxicity was not observed in any of the patients. After drug administration, there was no change in blood pressure and no deterioration in the post-operative course. In addition, there appeared to be no enhancement of IORT effects on normal tissue. However, there was a complication due to misadministration. In one patient with osteosarcoma, the entire solution of 7 g m$^{-2}$ KU-2285 was erroneously injected into the trachea; his arterial oxygen pressure dropped to 60 mmHg. After bronchoscopic suction of part of the solution, the patient's condition recovered in about 45 min and there were no long-term effects.

### Pharmacokinetics

Figure 1 shows plasma concentrations of KU-2285 after intrajejunal administration of 4.5, 6.8–7 or 9 g m$^{-2}$ of the compound in the patients with pancreatic or gastric cancer. The plasma concentration of KU-2285 appeared to reach a peak after 0.5–1 h of intrajejunal administration in most patients and then it gradually decreased. Figure 2 shows the peak plasma concentrations and tumour concentrations immediately after IORT of KU-2285 as a function of the administered dose in the 24 patients with pancreatic or gastric cancer. In three patients, tumour biopsy was not feasible. The average peak plasma concentration was 136 µg ml$^{-1}$ at the dose of 4.5 g m$^{-2}$, 215 µg ml$^{-1}$ at 6.8–7 g m$^{-2}$, and 301 µg ml$^{-1}$ at 9 g m$^{-2}$, and the average tumour concentration was 81, 166, and 333 µg g$^{-1}$ respectively. In the 21 patients with tumour biopsy, the average tumour–plasma ratio was 0.82 ± 0.32 (s.d.), although the true ratio may have been slightly higher because the tumour was biopsied only once in each patient.

Pharmacokinetic data for the five osteosarcoma patients are shown in Table 2 together with their treatment outcomes. In the patients receiving intragastric administration of KU-2285 in the supine position, the average peak plasma concentrations were 75–98% of those in the pancreatic or gastric cancer patients, in whom KU-2285 was administered into the upper jejunum. In three osteosarcoma patients, the timing of biopsy (and IORT) was delayed due to the rather complicated IORT procedure, so the exact tumour–plasma ratio was not evaluable. Interestingly, the patient in whom the drug was administered intratracheally showed high plasma levels of KU-2285.

One patient with bladder cancer receiving oral KU-2285 at 1 g m$^{-2}$ showed a peak plasma concentration of 15 µg ml$^{-1}$ 2 h after administration of the drug. Tumour biopsy could not be performed in this patient.

### Treatment outcome

Of the 22 patients with unresectable pancreatic cancer, 11 had no distant metastasis but 11 had liver metastasis and/or peritoneal dissemination. Figure 3 shows the survival curve for the 11 patients with localized unresectable tumours together with that for the 22 matched historical control patients. Although the difference between the KU-2285 group and the control group was not significant ($P$ = 0.26), the median survival time of 11 months for the

 © Cancer Research Campaign 1997

**Table 2** Characteristics of osteosarcoma patients treated with KU-2285 and IORT

| Age (years) | Sex | Site | KU-2285 dose (g $m^{-2}$) | KU-2285 level: Plasma[a] (μg $ml^{-1}$) [Time] | KU-2285 level: Tumour[b] (μg $g^{-1}$) [Time] | Local status | Status |
|---|---|---|---|---|---|---|---|
| 10 | F | Humerus | 3 | 65 [2 h] | 31 [3.5 h] | Control | 5 months, died of lung metastasis |
| 13 | M | Femur | 4.5 | 130 [2 h] | 33 [4.5] | Control 11 months prosthetic replacement | 43 months, died of intercurrent disease |
| 16 | M | Femur | 4.5 | 147 [2 h] | 91 [3 h] | Control 9 months prosthetic replacement | 38 months, no evidence of disease |
| 14 | M | Humerus | 4.5 | 104 [1 h] | 41[c] [2 h] | Control | 34 months, no evidence of disease |
| 52 | M | Tibia | 7[d] | 289 [2 h] | – | Control | 15 months, no evidence of disease |

[a]Peak plasma concentration; [b]tumour concentration at 10–20 min after completion of IORT; [c]concentration in peritumoural fat tissue; [d]in this patient, KU-2285 was erroneously injected into the trachea.

**Figure 3** Survival curves for the 11 patients with unresectable but localized pancreatic cancer receiving KU-2285 plus IORT and external beam radiation (—) and for the 22 matched historical control patients (· · ·)

**Figure 4** Local control curves for the 11 patients with unresectable but localized pancreatic cancer receiving KU-2285 plus IORT and external beam radiation (—) and for the 21 matched historical control patients (· · ·). Ticks represent individual patients alive or dead without local recurrence former group compares favourably with that of 8 months for the latter. The 2-year survival rate was 18% vs 4.5%. One patient in the KU-2285 group died of intercurrent disease at 25 months without sign of recurrence. The 11 patients with distant metastasis had a median survival time of 4 months (range 3–12 months), which was similar to that (3.5 months) in the historical control patients treated with IORT alone (Shibamoto et al, 1996*b*).

Figure 4 shows the local control curves for the 11 patients with localized unresectable pancreatic cancer receiving KU-2285 and for the 21 historical control patients. Local status was not assessable in one of the historical control patients. The 1- and 2-year local control rates were 50% and 40% respectively for the KU-2285 group, and both were 28% for the control group ($P = 0.10$). In the 11 patients with distant metastasis, the actuarial local control rate at 6 months was 44%.

There has been no local recurrence in the five patients with osteosarcoma, although two of them underwent ceramic prosthesis replacement of the irradiated tumour 9–11 months later because of pathological fracture (Table 2). Fracture of the irradiated tumour site is a common sequela of this treatment modality (Yamamuro et al, 1991). One patient with gastric cancer died of peritonitis carcinomatosa 1 year later. One patient with non-curatively resected pancreatic cancer is alive with high tumour marker levels but without evidence of local recurrence at 15 months after IORT. One patient with bladder cancer attained local control until 5 months, but then he was lost to follow-up.

### DISCUSSION

It is well known that hypoxic cell sensitizers are most effective when they are combined with single high-dose irradiation. Accordingly, misonidazole and etanidazole have been investigated in combination with IORT. Tepper et al (1987) used misonidazole at a dose of 3.5 g $m^{-2}$ in combination with IORT of 15–20 Gy for localized unresectable pancreatic cancer. They compared 41 patients receiving misonidazole with 22 historical control patients treated without misonidazole, but they did not find any significant difference between the two groups in either the survival rate or local control rate. This was a non-randomized comparison, and the control group had smaller tumours than the misonidazole group.

Indeed, the control group had a median survival time of 16.5 months, which is the longest one reported, so far, for unresectable pancreatic cancer (Shibamoto et al, 1996*c*). Nevertheless, the 2-year local control rate, as assessed by criteria similar to those used in this study, in the misonidazole group (45%) was higher, though not significantly so, than that in the control group (31%). Early development of distant metastasis in most patients was considered to obscure the possible benefit of misonidazole. For etanidazole, only the results of a phase I study have been reported to date (Halberg et al, 1994), and in that study most patients underwent IORT after resection of primary tumours, for which reason no information is available as to the efficacy of etanidazole. A 2-nitroimidazole nucleoside analogue RK-28 was tested in Japan in combination with IORT for mainly pancreatic cancer patients, but the study was discontinued before accrual of a sufficient number of patients to assess its efficacy (Sasai et al, 1992). A phase I trial for unresectable pancreatic cancer of another 2-nitroimidazole nucleoside analogue, PR-350 (Oya et al, 1995), is now on-going in Japan, but results regarding its efficacy are not yet available. Thus, no definite conclusions have been drawn as to the efficacy of hypoxic cell sensitizers when combined with IORT.

Apparently, KU-2285 has characteristics different from etanidazole. Owing to its fluorination, it has higher sensitizing activity, although its toxicity is similar. Because of its relatively high lipophilicity, faster and better distribution of the compound throughout tumour tissue can be expected. Therefore, we thought that KU-2285 was worthy of clinical evaluation. As this drug was originally intended to be given exclusively orally, no intravenous toxicity studies in large animals have been carried out and no pharmaceutical examination has been performed to allow its intravenous use. Therefore, we administered the compound into the jejunum or stomach, or orally. As partly expected from the results of the phase I study in combination with conventional radiotherapy, in which cumulative doses up to 28 g $m^{-2}$ were tolerable (Shibamoto et al, 1996*a*), we observed no toxicity of this compound up to the dose of 9 g $m^{-2}$. Because of the limited solubility, we did not increase the dose further, but considered 7 g $m^{-2}$ as the standard dose. These doses are lower than the maximum dose of 12 g $m^{-2}$ for etanidazole, but considering the aforementioned characteristics of KU-2285, further studies of this compound seem to be warranted.

Sufficiently high concentrations of KU-2285 to obtain definite radiosensitization were achieved when it was injected both into the jejunum and into the stomach, although the levels appeared slightly lower in the latter. The plasma levels of KU-2285 at 6.8–9 g $m^{-2}$ were higher than those of misonidazole (100 μg $ml^{-1}$ or higher) obtained in the IORT study (Tepper et al, 1987), but lower than the levels of etanidazole (700–1500 μg $ml^{-1}$) obtained at the dose of 12 g $m^{-2}$ (Halberg et al, 1994). This is partly because etanidazole was given intravenously. However, the distribution of KU-2285 into the tumour tissues was satisfactory. Although the true tumour–plasma ratio may not have been determined, the average ratio in pancreatic or gastric cancer patients was at least 0.82, which is higher than that (0.54) reported for etanidazole (Halberg et al, 1994). The mean tumour concentration of KU-2285 was 166 μg $g^{-1}$ after administration of 6.8–7 g $m^{-2}$ and 333 μg $g^{-1}$ after 9 g $m^{-2}$. At these concentrations, sensitizer enhancement ratios of at least 1.8 can easily be expected (Kagiya et al, 1989).

Although a definite conclusion cannot yet be drawn as to the efficacy of KU-2285 combined with IORT, the survival and local control rates in the 11 patients with localized unresectable pancreatic cancer compare favourably with those in our historical control patients. The patient–tumour characteristics and the IORT/external beam radiotherapy methods were similar in the two groups. Curing such patients is still difficult, but it may be possible to decrease or delay local recurrence by adding KU-2285 to IORT. We will continue to use IORT with KU-2285 for unresectable pancreatic cancer, as this treatment modality is not aggressive and long-term survival is occasionally achieved. In the previous study of misonidazole and IORT for unresectable pancreatic cancer (Tepper et al, 1987), the IORT dose was 15–20 Gy, whereas we used 30 Gy. In view of the higher doses of both the sensitizer and radiation, the conditions in our study would have been more suitable to investigating the efficacy of hypoxic cell sensitizers. In the five patients with osteosarcoma, we used a 60-Gy dose, which is usually sufficient to control osteosarcoma lesions (Yamamuro et al, 1991), and we found no local failure. In future, it may be possible to reduce this dose by the use of this compound.

In summary, we found no toxicity of KU-2285, when given before IORT, up to a dose of 9 g $m^{-2}$. Our preliminary results in unresectable pancreatic cancer appear encouraging. Proceeding to the next step seems to be appropriate. We are planning to combine KU-2285 not only with IORT but also with radiosurgery.

### ACKNOWLEDGEMENTS

We wish to thank Drs S Nishimoto, K Nakamura, Y Kotoura, M Takahashi, M Hiraoka, J Toguchida and K Sasai for valuable help in this study.

### REFERENCES

Adams GE (1978) Hypoxic cell sensitizers for radiotherapy. *Int J Radiat Oncol Biol Phys* **4**: 135–141

Brown JM (1995) Hypoxic cell radiosensitizers: the end of an era? Regarding Lee et al., IJROBP 32: 567–576, 1995. *Int J Radiat Oncol Biol Phys* **32**: 883–885

Coleman CN, Wasserman TH, Urtasun RC, Kalsey J, Noll L, Hancock S and Phillips TL (1990) Final report of the Phase I trial of the hypoxic cell radiosensitizer SR 2508 (etanidazole) Radiation Therapy Oncology Group 83–03. *Int J Radiat Oncol Biol Phys* **18**: 389–393

Dische S, Chassagne D, Hope-Stone HF, Dawes PJDK, Roberts JT, Yosef H, Bey P, Horiot JC, Jacobson A, Frankendal B, Gonzales Gonzales D, Nguyen TD, Daly NJ, Le Floch O, Newman H, Vieiro E, Bennett MH, Bichel P, Duvillard P, Cook PA, Everett V and Machin D (1993) A trial of Ro 03-8799 (pimonidazole) in carcinoma of the uterine cervix: an interim report from the Medical Research Council Working Party on advanced carcinoma of the cervix. *Radiother Oncol* **26**: 93–103

Halberg FE, Cosmatis D, Gunderson LL, Noyes RD, Hanks GR, Buswell L, Nagorney DM and Coleman CN (1994) RTOG #89–06: a Phase I study to evaluate intraoperative radiation therapy and the hypoxic cell sensitizer etanidazole in locally advanced malignancies. *Int J Radiat Oncol Biol Phys* **28**: 201–206

Kagiya T, Nishimoto S, Shibamoto Y, Wang J, Zhou L, He YL, Sasai K, Takahashi M and Abe M (1989) Importance of tumor affinity of nitroazoles in hypoxic radiosensitization. *Int J Radiat Oncol Biol Phys* **16**: 1033–1037

Lawton CA, Coleman CN, Buzydlowski JW, Forman JD, Marcial VA, DelRowe JD and Rotman M (1996) Results of a Phase II trial of external beam radiation with etanidazole (SR 2508) for the treatment of locally advanced prostate cancer (RTOG protocol 90–20). *Int J Radiat Oncol Biol Phys* **36**: 673–680

Lee DJ, Cosmatos D, Marcial VA, Fu KK, Rotman M, Cooper JS, Ortiz HG, Beitler JJ, Abrams RA, Curran WJ, Coleman CN and Wasserman TH (1995) Results of an RTOG Phase III trial (RTOG 85–27) comparing radiotherapy plus etanidazole with radiotherpay alone for locally advanced head and neck carcinomas. *Int J Radiat Oncol Biol Phys* **32**: 567–576

Overgaard J (1994) Clinical evaluation of nitroimidazoles as modifiers of hypoxia in solid tumors. *Oncol Res* **6**: 509–518

Oya N, Shibamoto Y, Sasai K, Sugiyama T and Abe M (1993) *In vivo* radiosensitization efficacy of KU-2285 and etanidazole at clinically relevant low radiation doses. *Int J Radiat Oncol Biol Phys* **27**: 1113–1119

 © Cancer Research Campaign 1997

Oya N, Shibamoto Y, Sasai K, Shibata T, Murata R, Takagi T, Iwai H, Suzuki T and Abe M (1995) Optical isomers of a new 2-nitroimidazole nucleoside analog (PR-350 series): radiosensitization efficiency and toxicity. *Int J Radiat Oncol Biol Phys* **33**: 119–127

Sasai K, Nishimoto S, Shimokawa K, Hisanaga Y, Kitakabu Y, Shibamoto Y, Zhou L, Wang J, Takahashi M, Kagiya T and Abe M (1991) A fluorinated 2-nitroimidazole, KU-2285, as a new hypoxic cell radiosensitizer. *Int J Radiat Oncol Biol Phys* **20**: 1249–1254

Sasai K, Shibamoto Y, Manabe T, Baba N, Takahashi M, Sakaguchi M and Abe M (1992) Pharmacokinetics of intratumoral RK-28, a new hypoxic radiosensitizer. *Int J Radiat Oncol Biol Phys* **24**: 959–963

Shibamoto Y, Nishimoto S, Shimokawa K, Hisanaga Y, Zhou L, Wang J, Sasai K, Takahashi M, Abe M and Kagiya T (1989) Characteristics of fluorinated nitroazoles as hypoxic cell radiosensitizers. *Int J Radiat Oncol Biol Phys* **16**: 1045–1048

Shibamoto Y, Streffer C, Sasai K, Oya N and Abe M (1992) Radiosensitization efficacy of KU-2285, RP-170 and etanidazole at low radiation doses: assessment by *in vitro* cytokinesis-block micronucleus assay. *Int J Radiat Biol* **61**: 473–478

Shibamoto Y, Takahashi M and Abe M (1996*a*) A phase I study of a hypoxic cell sensitizer KU-2285 in combination with conventional radiotherapy. *Radiother Oncol* **40**: 55–58

Shibamoto Y, Manabe T, Ohshio G, Sasai K, Nishimura Y, Imamura M, Takahashi M and Abe M (1996*b*) High-dose intraoperative radiotherapy for unresectable pancreatic cancer. *Int J Radiat Oncol Biol Phys* **34**: 57–63

Shibamoto Y, Nishimura Y and Abe M (1996*c*) Intraoperative radiotherapy and hyperthermia for unresectable pancreatic cancer. *Hepato–Gastroenterology* **43**: 326–332

Shibata T, Shibamoto Y, Oya N, Sasai K, Murata R, Ishigaki T and Abe M (1994) Comparison of radiosensitizing effect of KU-2285 and SR-2508 at low drug concentrations and doses. *Int J Radiat Oncol Biol Phys* **29**: 587–590

Tepper JE, Shipley WU, Warshaw AL, Nardi GL, Wood WC and Orlow EL (1987) The role of misonidazole combined with intraoperative radiation therapy in the treatment of pancreatic carcinoma. *J Clin Oncol* **5**: 579–584

Urtasun RC, Coleman CN, Wasserman TH and Phillips TL (1984) Clinical trials with hypoxic cell sensitizers: time to retrench or time to push forward? *Int J Radiat Oncol Biol Phys* **10**: 1691–1696

Yamamuro T, Kotoura Y, Kasahara K, Iwasaki R, Takahashi M and Abe M (1991) Limb salvage with intraoperatively irradiated tumor tissues preserved *in situ* in osteosarcoma. In: *Limb Salvage. Major Reconstructions in Oncologic and Nontumoral Conditions*, Langlais F and Tomeno B (eds), pp 619–625. Springer: N Verlag: Berlin

© Cancer Research Campaign 1997